# Misprediction of Structural Disorder in Halophiles

**DOI:** 10.3390/molecules24030479

**Published:** 2019-01-29

**Authors:** Rita Pancsa, Denes Kovacs, Peter Tompa

**Affiliations:** 1 Institute of Enzymology, Research Centre for Natural Sciences of the Hungarian Academy of Sciences, 1117 Budapest, Hungary; 2 VIB Center for Structural Biology (CSB), 1050 Brussels, Belgium; dkovacs@vub.ac.be; 3 Structural Biology Brussels (SBB), Vrije Universiteit Brussel (VUB), 1050 Brussels, Belgium

**Keywords:** extremophile, halophile, structurally disordered, intrinsically disordered, disorder prediction, sequence composition bias, adaptation, charge distribution

## Abstract

Whereas the concept of intrinsic disorder derives from biophysical observations of the lack of structure of proteins or protein regions under native conditions, many of our respective concepts rest on proteome-scale bioinformatics predictions. It is established that most predictors work reliably on proteins commonly encountered, but it is often neglected that we know very little about their performance on proteins of microorganisms that thrive in environments of extreme temperature, pH, or salt concentration, which may cause adaptive sequence composition bias. To address this issue, we predicted structural disorder for the complete proteomes of different extremophile groups by popular prediction methods and compared them to those of the reference mesophilic group. While significant deviations from mesophiles could be explained by a lack or gain of disordered regions in hyperthermophiles and radiotolerants, respectively, we found systematic overprediction in the case of halophiles. Additionally, examples were collected from the Protein Data Bank (PDB) to demonstrate misprediction and to help understand the underlying biophysical principles, i.e., halophilic proteins maintain a highly acidic and hydrophilic surface to avoid aggregation in high salt conditions. Although sparseness of data on disordered proteins from extremophiles precludes the development of dedicated general predictors, we do formulate recommendations for how to address their disorder with current bioinformatics tools.

## 1. Introduction

The recent recognition of intrinsically disordered proteins and regions (IDPs/IDRs) that lack a well-defined structure under physiological conditions [1,2,3,4] revolutionized structural biology research. Such proteins perform their functions as conformational ensembles and are prevalent in most organisms, with eukaryotes typically having more than prokaryotes [5,6]. Based on diverse biophysical techniques, we have conclusive evidence for the structural disorder of 1539 protein regions within 694 proteins, which are deposited in the DisProt database (release 6.02) [7]. This database provides accurate residue-level assessment of the order/disorder status of proteins and served as a training set for several dedicated predictors [8,9].

**Figure 1:**
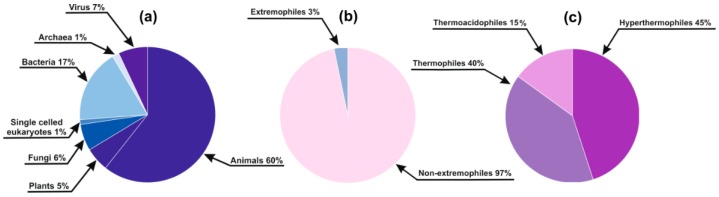
Species distribution of the DisProt Database: (**a**) Distribution of DisProt entries among large phylogenetic groups; (**b**) Distribution of the represented species among extremophiles and non-extremophiles; (**c**) Distribution of the 20 extremophilic proteins among different lifestyle extremities.

Since experimental data on disorder in DisProt cover only a tiny fraction of (even the best covered human) proteome, many of our basic concepts and generalizations of protein disorder rely on bioinformatics predictions, including its phylogenetic distribution and evolution [5,6]. However, it has never been directly addressed how much we can extrapolate our conclusions to species outside those covered in DisProt. That is, a potentially serious and thus far unappreciated limitation of applying predictors is that they have all been trained on disordered proteins from organisms of a rather limited phylogenetic circle and range of habitats. Due to these reasons, any type of evolutionary adaptation causing systematic compositional bias in protein sequences could potentially mislead the prediction methods. For instance, proteins of (micro)organisms living under extreme environmental conditions (extremophiles) are under severe challenge not to lose their structural integrity [10,11,12,13,14,15,16,17,18]. Extremophile sequences are practically absent from DisProt (Figure 1), which raises the question of whether they can be accurately handled by disorder prediction algorithms, i.e., how much we should trust such prediction results.

Although several prior observations caution that there might be systematic prediction errors due to these reasons [19,20,21,22], those have never been directly addressed. For example, when structural disorder was addressed in Archaea and in extremophiles, very high levels of disorder were reported for halophiles and alkaliphiles [20,22], and interpreted as a sign of increased need for complex regulation in them [22]. However, from the point of view of evolutionary adaptation, the saline lakes where most halophiles live represent relatively static environmental conditions coupled with a low number of possible competitors, which do not imply the increased need for complex regulatory mechanisms and consequently structural disorder. Additionally, Archaea are consistently predicted to have less disorder than Bacteria, but it has never been noted that this could well be due to their significant bias for hyperthermophilic species [16] and not due to their phylogenetic identity *per se*. Although biased amino acid composition is the primary reason for structural disorder [23,24,25], and proteins of extremophiles do possess unusual amino acid composition [10,26,27], these two are based on different underlying reasons and therefore should be carefully separated and their relationship should be precisely analyzed before interpreting any prediction results gained for such sequences.

Here we investigate this phenomenon by applying disorder predictors to the proteomes and individual proteins of extremophiles and comparing their predicted disorder to those of mesophiles. We report systematic prediction errors in a particular extremophile group, explain how the observed tendencies make biological sense, and formulate some general recommendations to arrive at a more realistic picture when using the available methods on their sequences.

## 2. Results

### 2.1. Species Distribution of the DisProt Database

In the DisProt Database (release 6.02) [7], which served as a training set for most disorder prediction methods, only 20 sequences (<3% of the database) belong to species living among extreme conditions (10 Archaea and 10 Bacteria; nine hyperthermophiles and eight thermophiles, with three of them also displaying acidophilic characteristics (thermoacidophiles); Figure 1). Thus, we can conclude that extremophile groups are hardly or not at all represented in DisProt, which precluded the training of any methods that could distinguish them from other DisProt sequences.

### 2.2. Assessing Structural Disorder in Extremophile Groups

**Figure 2:**
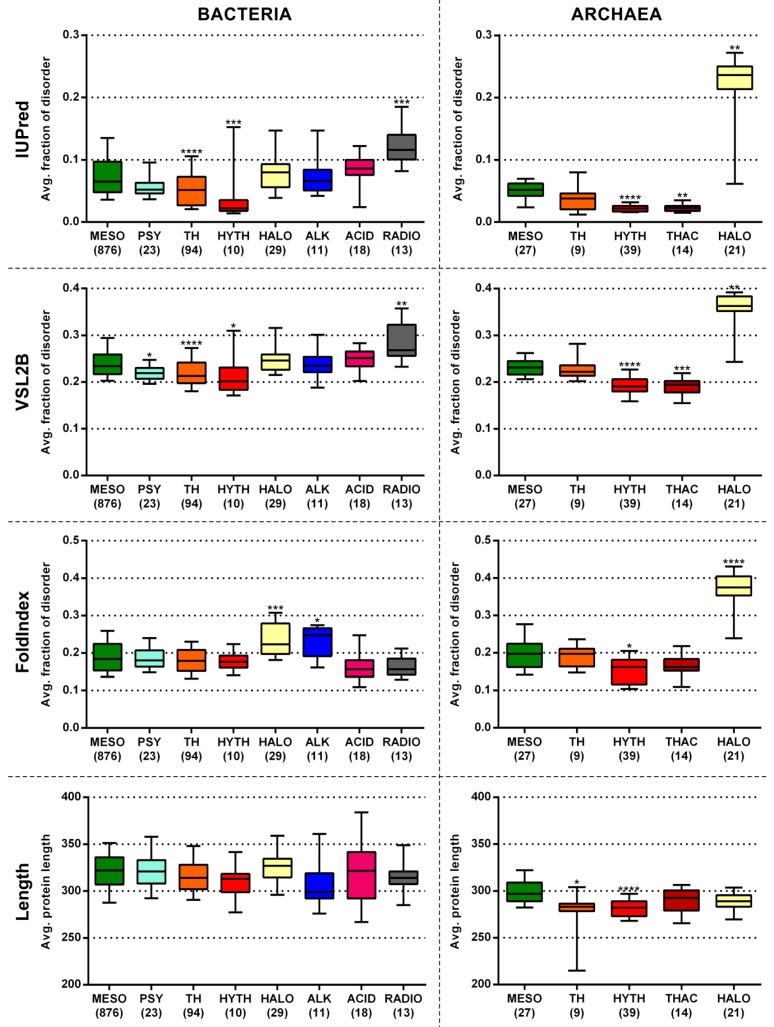
Predicted disorder content of Bacteria and Archaea with different lifestyles. The average fraction of disordered residues was calculated for each proteome by three prediction methods. The distribution of the calculated values of each lifestyle group was compared to that of mesophiles by Dunn’s multiple comparison test for Bacteria and Archaea separately. The significance levels of the distribution differences are indicated as stars above the boxes (* *p* < 0.05; ** *p* < 0.01; *** *p* < 0.001; **** *p* < 0.0001). The number of proteomes in the different lifestyle groups are indicated below the labels. MESO—mesophiles, PSY—psychrophiles, TH—thermophiles, HYTH—hyperthermophiles, HALO—halophiles, ALK—alkaliphiles, ACID—acidophiles, RADIO—radiotolerants, THAC—thermoacidophiles.

We calculated the mean fractions of disordered residues for the proteomes belonging to each extremophile lifestyle group and compared their distributions to that of the mesophilic reference group for Bacteria and Archaea, separately, with three prediction methods (see further details on these predictors in Materials and Methods) (Figure 2). We found similar tendencies as Vicedo et al. [20]. Average protein length was also compared between the different groups and mesophiles (as reference); moreover, the comparisons were repeated using only protein subsets with annotated Enzyme Commission (EC) numbers (by UniProt [28]; Figure S1), hereafter referred to as enzymes, to see if the observed deviations were due to the addition or loss of disordered proteins/regions or if they were rather due to an adaptive change generally affecting the compositions of all proteins (including enzymes), without necessarily affecting their structural status. All calculated data are presented in Tables S1 and S2 for Bacteria and Archaea, respectively.

The three prediction methods provided similar tendencies among the lifestyle groups, even though they showed remarkable differences in the absolute values of predicted disorder. Additionally, full proteomes and enzymes showed very similar tendencies with each method, with enzymes having lower predicted disorder in absolute terms (Figure 2 and Figure S1).

Acidophilic and alkaliphilic bacteria did not show significant disorder deviations from mesophiles, which is in line with the fact that they maintain circumneutral intracellular pH through diverse pH homeostasis mechanisms [29,30], and therefore did not need to extensively adapt the compositions of their intracellular proteins to their environment.

Thermophiles and hyperthermophiles had significantly lower predicted disorder with almost all prediction methods. Among Archaea there are many hyperthermophiles but few thermophiles, so for the former a statistically stronger comparison could be made, while for the latter the comparison was too weak to show significant difference. Among Bacteria, both the many thermophiles and few hyperthermophiles showed significantly lower disorder content than mesophiles with two of the three methods. Therefore, if there are sufficient numbers of proteomes to compare, both thermophiles and hyperthermophiles show a significant reduction in disorder content, with hyperthermophiles having more extreme values. Actually, the physical basis of protein stability in (hyper)thermophiles has been analyzed in great detail in the case of folded proteins, leading to the general picture that extended H-bond and electrostatic networks on the surface, better atomic packing, and reduced surface dynamics all contribute to increased thermal stability [29,30]. Whereas this effect can be rationalized and predicted, evolutionary strategies against other destabilizing physical and chemical factors have been studied in much less detail and are thus poorly understood. The involvement of structural disorder in heat resistance has been addressed in one study only [31], leaving the question, if predicted reduced disorder is also a structural fact, open. Other extreme conditions have not yet been generally approached by either experiment or bioinformatics, i.e., our study is the first to address this question in a systematic manner.

Whether thermophilic and hyperthermophilic proteins are affected by a misprediction of disorder is very difficult to answer. The reason for this is that they hardly have any disordered regions whose predictions can be fairly compared to the corresponding mesophilic homologs because all of them have been lost or at least heavily shortened through evolution, probably for the sake of increasing thermal stability [31]. We have selected the five most closely related mesophile–hyperthermophile species pairs from our dataset (four bacterial and one archaeal, see Table 1), downloaded their 1:1 orthologous protein pairs from the OMA Orthology database, searched for all the ≥40 residues IUPred-predicted consecutive long disordered regions (LDRs) in the mesophilic species, aligned the orthologous pairs by ClustalW 2.0.12, identified the corresponding regions within the hyperthermophile orthologs using the alignments, and finally calculated how big percent of the length of the mesophilic LDRs have been preserved in them (Table 1). We found that on average only half of the identified LDRs preserved at least 75% of their length in the hyperthermophiles, with many of the well-preserved regions belonging to ribosomal proteins or matching the regions that we have previously identified as the most essential LDRs of prokaryotic housekeeping proteins, which are so strongly preserved in evolution that even minimal genomes (endosymbiotic bacteria that have undergone extreme genome reduction) have them [32].

**Figure 3:**
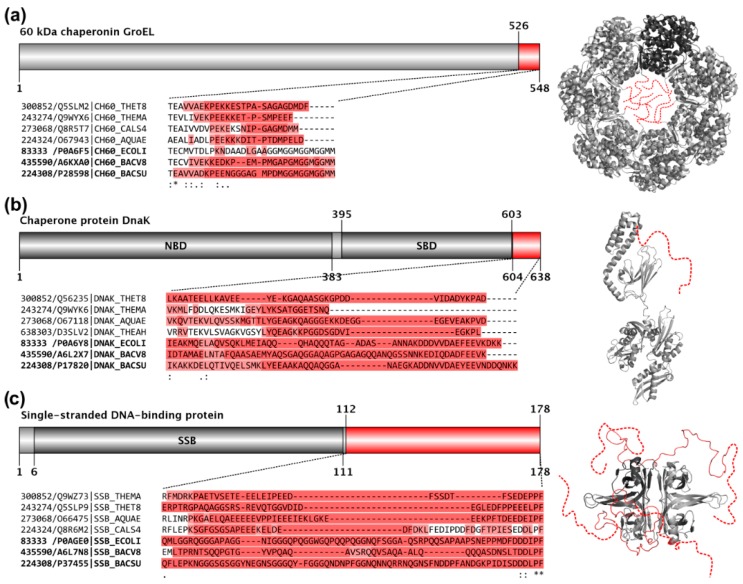
In hyperthermophiles, even essential IDRs are largely shortened. Domain maps, structures, aligned sequences, and IUPred-predicted disordered segments are depicted for three essential proteins, GroEL (**a**), DnaK (**b**), and SSB (**c**), with conserved disordered *C*-terminal regions that likely exist and function as disordered ensembles in all species [32]. On the grey domain maps, the residue boundaries of conserved disordered segments (in red) and known domains (in darker grey) are provided for the *E. coli* K12 protein. The red regions of the domain maps complemented by a few residue positions are highlighted as Clustal Omega 1.2.2. multiple sequence alignments below the domain maps. The sequences of the organisms are identified by their Taxonomy/UniProt identifiers. In each case there are four hyperthermophilic sequences shown on the top and three mesophilic reference sequences bellow (identifiers in bold) as a reference. In the alignments the background of the residues are colored according to the corresponding IUPred predictions; residues with a score >0.5 have darker red background, residues with a score between 0.5 and 0.4 have lighter red background, while residues with a score <0.4 have no background. The *E. coli* structures of the corresponding proteins, GroEL (Protein Data Bank(PDB) ID:2NWC), DnaK (PDB ID:2KHO), and SSB (PDB ID:1QVC), are also depicted in light grey with the conserved disordered segments marked by red or added as red dashed lines (or as the combination of the two). For a better understanding of the correspondences, in both the heptameric GroEL and tetrameric SSB structures, one chain is depicted by darker grey than the others.

We checked the presence of the previously independently obtained non-ribosomal obligate LDRs in some well-known hyperthermophilic organisms and compared them to reference mesophilic organisms. Such examples include the *C*-terminal tails of GroEL, DnaK, and the single-stranded DNA-binding protein, SSB. It turns out that in hyperthermophiles even these absolutely essential IDRs have been largely shortened, i.e., when aligning these segments with their mesophilic homologs, the hyperthermophilic sequences are heavily gapped (Figure 3), even though the folded fractions of the same proteins are well-preserved. These examples illustrate well that the very low predicted disorder content of these extremophile proteomes is definitely mainly due to the loss of IDRs. Since hyperthermophiles live at not only extreme, but very static habitats with hardly any competition, their gene sets have undergone a severe reduction to only 2000 ± 500 protein-coding genes in both Bacteria and Archaea, which is significantly less than those of mesophilic species (Bacteria *p* = 0.0222; Archaea *p* = 0.0160). This process went along a radical erosion of their remaining proteins (Figure 2 and Figure S1), i.e., the average protein length in hyperthermophilic Archaea is significantly shorter than in mesophiles (*p* < 0.0001; Figure 2). This erosion mostly affected their IDRs, which were probably thermodynamically unfavorable and provided complex regulatory possibilities, which may be superfluous in such static environments.

Psychrophile bacteria did not show significant disorder deviations from mesophiles. This might be due to the fact that the few organisms belonging here represent a mixture of real psychrophiles and psychrotolerants, which can grow in cold environments but do not require them.

There are two groups for, which we observed significantly higher predicted disorder content than for mesophiles: radiotolerants (among Bacteria) and halophiles (among both Bacteria and Archaea). The group of radiotolerants was largely made up by the *Deinococcus* genus, which is an ancient group with no clear relationship to any of the other known bacterial lineages [29]. *Deinococcus* species have several unique protein families as well as orphans and *Deinococcus*-specific proteins of unknown function [30,31]. Their extraordinary resistance to ionizing radiation, oxidative stress, desiccation, and other damaging conditions has never been attributed to an amino acid composition bias of their proteins, but rather to the action of specific DNA repair [29] and antioxidation systems, and transcription regulation during stress response [30].

Therefore, the high level of predicted disorder content in these proteomes is probably not a result of misprediction, but (1) the fact that many of their proteins contain long intrinsically disordered, often low complexity regions that are not present in non-extremophile homologues [31], and likely contribute to desiccation resistance [32]; (2) the increased presence of orphan proteins in each species [29] (orphan proteins have a high propensity for disorder in general [33,34]); and (3) the increased numbers of DNA repair and transcription regulation proteins that have relatively high levels of structural disorder in general [35,36].

**Figure 4:**
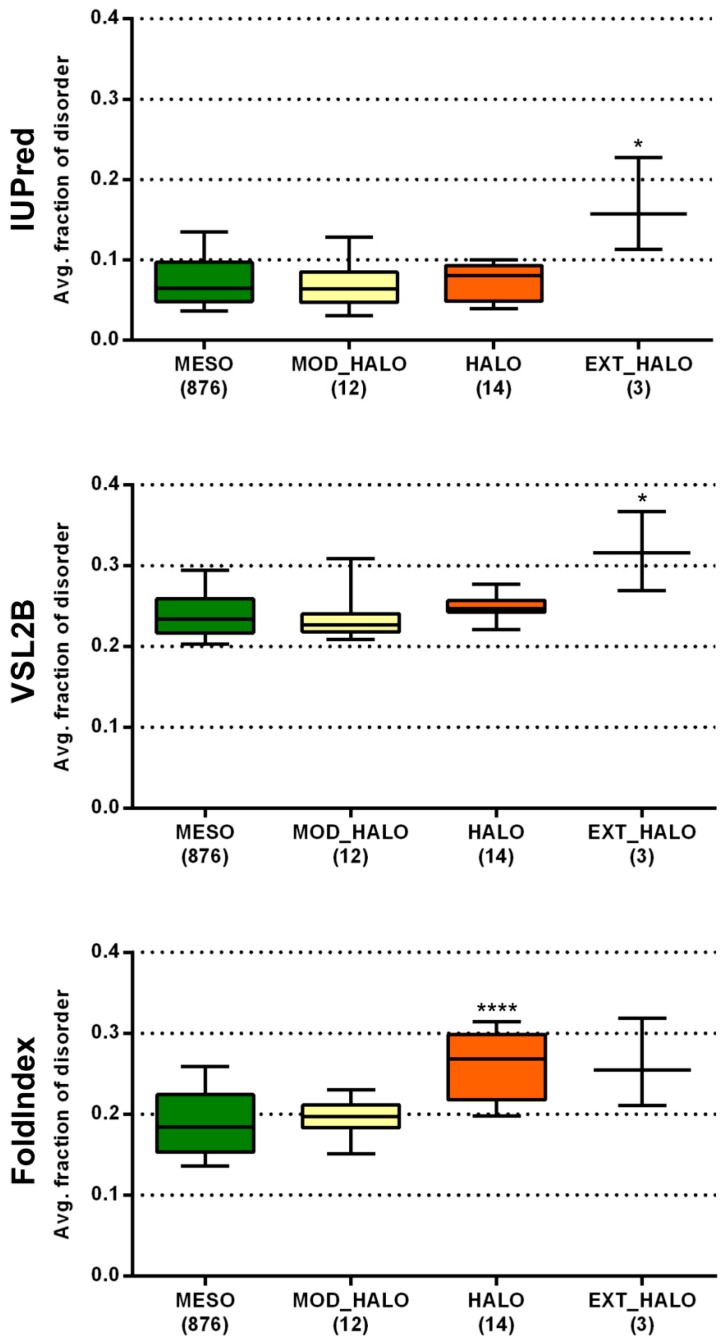
Predicted disorder content of different extremity classes of bacterial halophiles. The average fraction of disordered residues was calculated for each proteome by three prediction methods. The distribution of the calculated values of each class was compared to that of mesophiles by Dunn’s multiple comparison test, with the significance levels indicated as stars above the boxes (* *p* < 0.05; **** *p* < 0.0001). MESO—mesophiles, MOD_HALO—moderate halophiles, HALO—halophiles, EXT_HALO—extreme halophiles.

Halophilic Bacteria grow over an extended range of salt concentrations (3–20% NaCl, *w*/*v* and above), with many being only moderately halophilic, unlike the obligate halophilic Archaea whose growth is restricted to high saline environments [33]. This could be the reason why archaeal halophiles had uniformly very high disorder content with all three prediction methods (higher than any other lifestyle group), while bacterial halophiles were only found to be significantly more disordered than mesophiles with FoldIndex. To see if there is a relationship between the level of salt requirement/tolerance of bacterial halophiles and their predicted proteome-level disorder content, we classified the respective 29 species into three groups, moderately halophilic, halophilic, and extremely halophilic (see further details on this classification in Materials and Methods and in Table S3), and compared their predicted disorder content with all three prediction methods to that of mesophiles (Figure 4). While moderately halophilic species were not different from any of the three prediction methods, halophilic species were more disordered than mesophiles with FoldIndex and extremely halophilic species with both IUPred and PONDR VSL2B. The largely elevated disorder levels seen for extreme halophiles among both Bacteria and Archaea are due to a completely different reason than in the case of radiotolerants. Since halophiles usually live in static environments, it is very unlikely that they would benefit from maintaining an increased complexity of regulatory mechanisms that would require the excessive involvement of disordered proteins/regions. Halophilic Bacteria and Archaea were also reported to have some large unstructured hydrophilic low complexity protein regions that may provide them with resistance to dehydration [34]. This is in line with the suggested chaperone effect of IDPs, such as late embryogenesis abundant (LEA) proteins and dehydrins [35,36], which appears as a widespread stress-related protective mechanism in diverse organisms such as bacteria [34], plants [37], or animals [38]. Still, this is probably not the primary reason for the high predicted disorder in halophiles; rather, it is due to their unusually high net negative surface charge, i.e., an increase in Asp and Glu residues and a decrease in nonpolar and basic surface residues, which may help them avoid aggregation in high salt conditions [26,27]. The proteomes as well as the IUPred-predicted disordered regions of halophiles have significantly more acidic residues than those of mesophiles for both Bacteria and Archaea (Figure S2). We propose that the high net negative surface charge misleads disorder prediction methods, especially the ones that are purely based on sequence, so that they predict even well-folded halophilic proteins/enzymes to be disordered.

### 2.3. Halophile–Mesophile Protein Homologs with Available Structures Clearly Support Overprediction of Disorder in Halophiles

**Figure 5:**
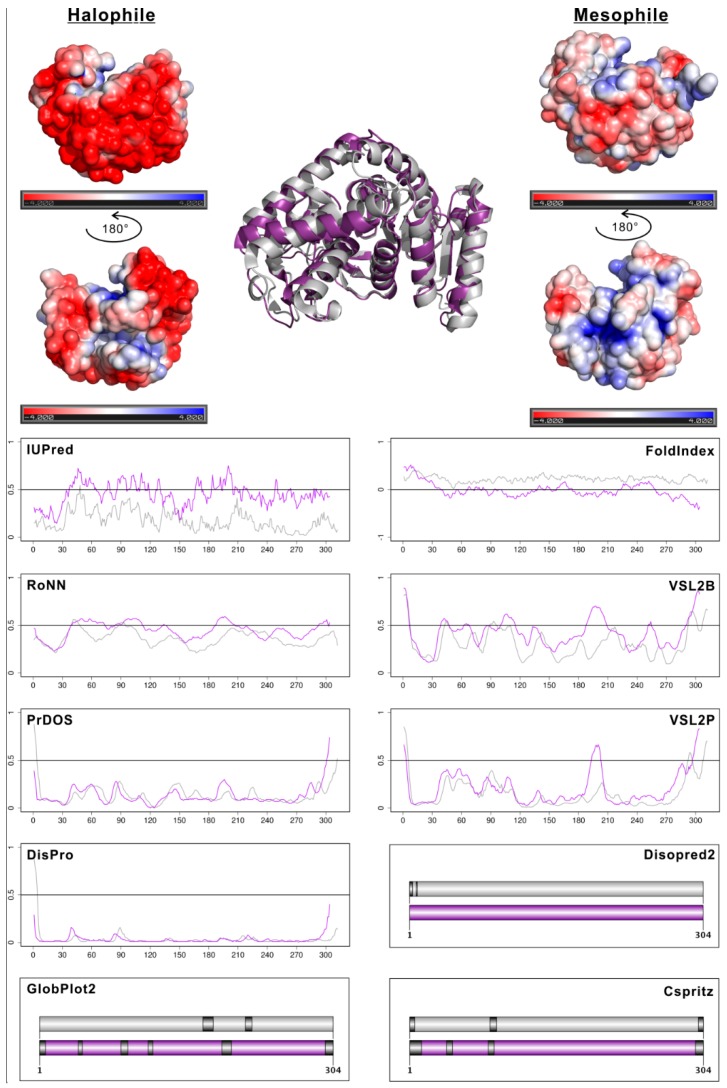
Disorder predictions with ten methods for a halophilic and a mesophilic malate–dehydrogenase enzyme. The halophilic enzyme from *Haloarcula marismortui* (PDB:1D3A) is shown on the left and with purple color in the structural alignment and within the disorder prediction plots. The mesophilic enzyme from *Bacillus anthracis* (PDB:3TL2) is shown on the right and with grey color in the structural alignment and within the disorder prediction plots. For the different disorder prediction methods, order–disorder thresholds were defined as suggested in their respective original publications. In each case values are above the probability threshold mean disorder, except for FoldIndex where it is the opposite. For the seven methods that provide continuous values for disorder probability we show the predicted values as curves. The curves are continuous and just placed on each other, not fitted/aligned. Purple curves show the predictions for the halophilic, grey ones for the mesophilic enzyme. For the three methods that only provide a binary classification of residues as ordered/disordered we show the proteins as colored bars with the predicted disordered regions depicted as dark grey stripes and with the length of the halophilic protein indicated.

To investigate the role of high net negative surface charge of halophilic proteins, we decided to select a few enzymes that have both an extreme halophile and a mesophile version in the Protein Data Bank (PDB) with high sequence- and structure-identity and similar length. Since there are not many of such pairs and it is difficult to perform a targeted search, we show here three examples that we could identify in a relatively short time. These include a pair of malate-dehydrogenase enzymes from *Haloarcula marismortui* (halophile; PDB:1D3A) and *Bacillus anthracis* (mesophile; PDB:3TL2) (Figure 5), a pair of nucleoside diphosphate kinase enzymes from *Halobacterium salinarum* (halophile; PDB:2AZ3) and *Staphylococcus aureus* (mesophile; PDB:3Q86) (Figure S3), and a pair of catalase peroxidase enzymes from *H. marismortui* (halophile; PDB:1ITK) and *Mycobacterium tuberculosis* (mesophile; PDB:1SJ2) (Figure S4). Disorder predictions were carried out on these pairs by a wide variety of prediction methods, namely IUPred [39], FoldIndex [40], RONN [41], PONDR VSL2B [42], PrDOS [43], PONDR VSL2P [42], DisPro [44], Disopred2 [6], Globplot2 [45], and CSpritz [46]. To help understand the biophysical principles underlying the markedly elevated disorder profiles of the halophilic proteins we also showed their surface charge patterns. The marked differences between the surface charge patterns of homologous halophile–mesophile protein pairs have already been previously demonstrated for dihydrofolate reductase [47,48], malate-dehydrogenase [49], 2Fe-2S ferredoxin [50], proliferating cell nuclear antigen (PCNA) [51], manganese superoxide dismutase [52], elongation factor EF-Tu [53], and glutamate dehydrogenase [54].

In each presented and published case, along with high sequence- and structure-identity, a vast excess of negative charges was found on the surface of the halophilic protein. Proteome-wide computational analyses also supported the finding that this feature does not depend on the individual protein structures, but is applicable to all proteins encoded by halophilic organisms [55] and thus can be regarded as a general adaptive strategy to survive in high salt concentrations. For the three enzyme pairs depicted in Figure 5, Figure S3, and Figure S4 we saw markedly elevated predicted disorder values for the halophilic counterparts with IUPred, FoldIndex (here smaller values mean a larger propensity for disorder), VSL2B, and RONN, and somewhat elevated values with GlobPlot2. Thus, these purely sequence-based predictors are more prone to overestimate (mispredict) disorder due to the adaptive sequence composition bias typical for halophiles. However, the remaining methods that relied not only on sequence but also on phylogenetic information [55] mostly by generating PSI-BLAST profiles (PrDOS, VSL2P, DisPro, DISOPRED2, CSpritz) provided better results. When using PrDOS, DisPro DISOPRED2, or CSpritz, the differences between the predicted values for mesophiles and halophiles were negligible for all three protein pairs.

### 2.4. Archaea have Lower Disorder Levels than Bacteria due to Their Bias Towards Hyperthermophilic Species

**Figure 6:**
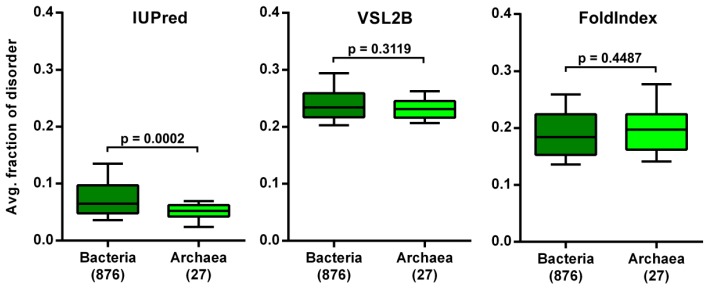
Comparison of the disorder levels of mesophilic Bacteria and Archaea. The average fraction of disordered residues was calculated for each proteome by three prediction methods and the resulting distributions were compared between Bacteria and Archaea by Mann–Whitney U tests, with p-values and the number of proteomes in the two groups indicated.

The disorder levels of the Kingdoms of living things have been compared many times by different research groups using different prediction methods [5,6,21]. The conclusions were always similar. Archaea had the lowest disorder level, significantly lower than Bacteria or Eukaryotes. What we observed here, however, by two of the three applied prediction methods, was that mesophilic archaea were not significantly less disordered than mesophilic bacteria (Figure 6). These results imply that Archaea (39/112, ~35% of hyperthermophilic species) are less disordered than Bacteria (10/1052, ~1%) due to their bias towards species living in extreme environments that necessitate such adaptive sequence composition changes, and not due to their phylogenetic identity *per se*.

## 3. Discussion

In this work we show that the evolutionary adaptation strategies of extremophile proteins to maintain their functions under demanding environmental conditions can mislead disorder prediction methods, especially the ones purely based on sequence, which do not usually face such sequence composition deviations during their training process.

For the three extremophile groups with significant deviations in their proteome-level disorder contents from those of mesophiles, we tried to determine the reasons underlying the observed tendencies. While hyperthermophiles and radiotolerants seem to lack [31] or amass disordered proteins and regions, respectively, as part of their adaptation strategies, in the case of halophiles the very high predicted disorder content is most probably a misprediction issue. Not just the whole proteomes but also the enzymes of halophiles were predicted with very high disorder content. Thus, the observed deviation could not be due to a general trend of employing additional disordered proteins or proteins being extended by disordered regions, but rather to all proteins being predicted with higher values due to their adaptive compositional biases. In halophiles there is an excess of acidic residues on the surface of proteins [48,56], because their interaction with the solvent can maintain a hydration layer that allows halophilic proteins to retain their functional state at molar salt concentrations (the residue–ion interaction model of halophilic protein stabilization) [57]. However, this adaptive compositional bias makes them look similar to intrinsically disordered proteins [56] in the eye of disorder prediction methods. This assumption was confirmed by analyzing halophile–mesophile protein pairs with available structures. Although halophile structures seemed essentially identical to their mesophile counterparts, they had a distinctive pattern of acidic surface charges, which led to significantly elevated predicted disorder tendencies. We thus confidently claim that the repeatedly reported high disorder content in halophiles [20,22] is a result of misprediction by multiple prediction methods, since their well-folded proteins were also predicted to be disordered. Therefore, halophilic organisms do not employ an increased complexity of regulatory mechanisms as proposed previously based on their high predicted disorder levels [22].

Based on our results, it is the lifestyle extremities of the majority of Archaea that lead to very low levels of predicted disorder. Almost all archaeal species live unusual lifestyles. Even the ones categorized as mesophilic are obligate or facultative anaerobe methanogen species living in sediments or wastewater. Importantly, there are many extremely hyperthermophilic (~35%), thermophilic (8%), and thermoacidophilic (12.5%) species among them, which lower their predicted proteome disorder levels. Sequenced extremely halophilic species that could counterbalance this effect by the observed overprediction make up only a smaller fraction of archaeal proteomes (~20%). Based on the observed data we suppose that if one could carefully select archaeal and bacterial species from the same environments and compare them, there would not be a significant difference between their disorder levels.

The structural state of proteins in general, and therefore also protein disorder, is largely dependent on environmental conditions (pH, salt, metals, cofactors, pressure, crowding, etc.). However, disorder predictors work based on the sequence, or other sequence-related more complex features (evolutionary information, predicted secondary structure, etc.), and thus are absolutely not able to relate to the above conditions. On top of this issue, the proteins of organisms facing extreme environmental conditions might have adaptive sequence compositional changes that make their folded proteins look similar to (mesophilic) IDPs. Since the amount of proven IDPs is insufficient to develop dedicated predictors for any group of extremophiles, we can only call for the cautious use of the current methods and formulate some general rules that can be applied to arrive at a more realistic picture when using them. Based on our experience with halophiles, second and third generation prediction methods taking into account evolutionary information, for instance, PrDOS, DisPro DISOPRED2, or CSpritz, are more likely to perform well on sequences with specific adaptive changes, so we would recommend using such slower, albeit more reliable tools on extremophile sequences.

## 4. Materials and Methods

The collections of representative bacterial and archaeal proteomes with 75% of co-membership threshold were downloaded from the PIR database (release 5.7) [58]. The resulting 1052 bacterial and 112 archaeal proteomes were grouped according to the lifestyle extremities of the corresponding species, which were assigned mainly by relying on the annotations in GOLD (Genomes OnLine Database) [59]. For Bacteria, 8 main lifestyle groups were created: mesophiles, thermophiles, hyperthermophiles, halophiles, acidophiles, alkaliphiles, psychrophiles, and radiotolerants. Species with an optimal growth temperature above 75 °C were considered to be hyperthermophiles, while those with 45 to 75 °C were grouped as thermophiles. Mixed groups (like thermoacidophiles) were not created because the increased fragmentation of the data could obscure important trends. If a given species showed more than one lifestyle extremity, it was put into each of the corresponding groups. For Archaea, 5 groups were created: mesophiles, halophiles, thermophiles, hyperthermophiles, and (hyper)thermoacidophiles, since the other pure extremity groups were not represented by a sufficient number of species (<5). Among Archaea there were many (hyper)thermoacidophiles, which is why we created a separate group for them. Except for those, if a given species showed more than one lifestyle extremity, it was put into each of the corresponding groups.

We searched for the optimal and maximally tolerated NaCl concentrations for the 29 halophilic bacterial species and classified them into three groups (Table S3). We accepted bacteria as extremely halophilic/halotolerant if they could grow in >30 *w*/*v*% NaCl. We classified them as halophilic if they required at least 3% NaCl for growth but could also tolerate 15–25%, and moderately halophilic if they required no or <10% NaCl and could not tolerate >15%.

Disorder was calculated for all the sequences in each proteome by three popular prediction methods: IUPred [39], PONDR^®^ VSL2B [42], and FoldIndex [40]. These methods were chosen based on their representation of different theoretical approaches, and because they allowed for the prediction of whole proteomes in a reasonable time. IUPred predicts structural disorder using sequence information alone, based on the possible pairwise interaction energies (calculated from precomputed statistic potentials for each possible amino acid pair) between a given residue and the residues of the surrounding sequence windows. If enough possible favorable interactions prevail with the given protein segment, the amino acid is predicted to be ordered, but if not, it is predicted to be disordered. PONDR^®^ VSL2B is a support vector machine that uses sequence composition features as inputs. FoldIndex takes into consideration the net charge and the sum of normalized hydrophobicity scale values (like charge-hydrophobicity plots) for a sequence window to distinguish between ordered and disordered residues.

The FoldIndex method was reprogrammed based on the simple formula provided in the original article and then the predicted scores were inverted on the order/disorder threshold value, so that the higher values meant more disorder, like in case of the other methods. This reinvented FoldIndex was used for the proteome-level analysis, while for the individual proteins in Figure 5, Figure S3, and Figure S4 the original values provided by the FoldIndex server were used.

Dunn’s multiple comparison tests were applied to evaluate the statistical significance of distribution differences when more than two datasets were compared (comparisons of multiple lifestyle groups to mesophiles), while Mann–Whitney U tests were used for pairwise comparisons (e.g., between the bacterial and archaeal mesophiles).

All data processing in this study was performed using custom Perl scripts (v5.10.1, Perl Foundation, Holland, MI, USA). All statistical analyses were implemented in GraphPad Prism 6 (GraphPad Software, San Diego, CA, USA). The Pymol molecular graphics tool (v1.2r2, Schrödinger, LLC, New York, NY, USA), the statistical analysis programming language R (R Foundation, Vienna, Austria), and Microsoft Excel 2010 (Microsoft Corporation, Redmond, WA, USA) were also used for figure preparation.

## Figures and Tables

**Table 1:** Orthologous IUPred-predicted consecutive long disordered regions (LDRs) between mesophilic and hyperthermophilic species.

Mesophile Species	Hyperthermophile Species	Number of 1:1 Protein Orthologs	Number of LDRs (≥40 res.) in Mesophile	Number of LDRs with ≥75% of Length Preserved (of which Ribosomal)
*Clostridium perfringens (ATCC 13124)*	*Caldicellulosiruptor saccharolyticus (ATCC 43494)*	717	15	6 (3)
*Clostridium perfringens (ATCC 13124)*	*Thermaerobacter marianensis (ATCC 700841)*	539	9	7 (3)
*Clostridium perfringens (ATCC 13124)*	*Thermoanaerobacter tengcongensis (DSM 15242)*	782	15	7 (3)
*Desulfitobacterium hafniense (DSM 10664)*	*Carboxydothermus hydrogenoformans (DSM 6008)*	1109	36	15 (3)
*Methanococcus maripaludis (S2)*	*Methanocaldococcus jannaschii (DSM 2661)*	1078	8	6 (4)

## Supplementary Materials

The following are available online.
