# Supplementary material for: Misprediction of Structural Disorder in Halophiles
Source: Molecules. 2019 Jan 29;24(3):479. doi: 10.3390/molecules24030479 (PMC6384707; doi:10.3390/molecules24030479)
Supplement: Supplementary file 1 [file molecules-24-00479-s001.zip › Supplementary_Information/Supplementary_figures.pdf]

Supplementary information for

Misprediction of structural disorder in halophiles

Rita Pancsa[1]*, Denes Kovacs[2] and Peter Tompa[1,2,3]*

[1] Institute of Enzymology, Research Centre for Natural Sciences of the Hungarian Academy of Sciences, 1117 Budapest, Hungary
[2] VIB Center for Structural Biology (CSB), 1050 Brussels, Belgium
[3] Structural Biology Brussels (SBB), Vrije Universiteit Brussel (VUB), 1050 Brussels, Belgium

* Correspondence: peter.tompa@vub.be; Tel.: +32 2 629 1924 (P.T.) and pancsa.rita@ttk.mta.hu; Tel.: +36 1 382 6705 (R.P.)

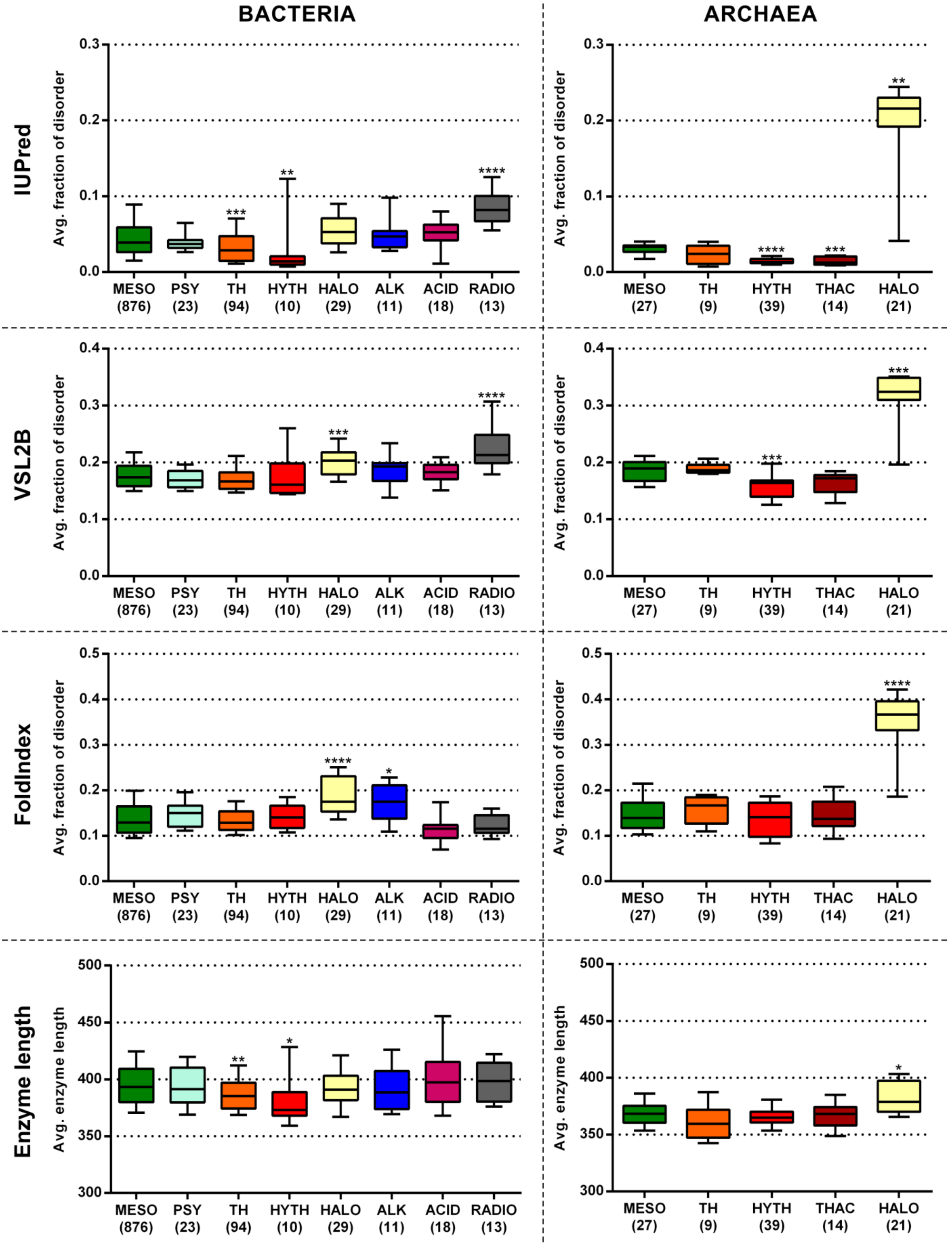

Figure S1. Predicted disorder content of Bacterial and Archaeal enzymes for different lifestyle groups. The average fraction of disordered residues was calculated for the enzymes of each proteome by three methods. The distribution of the calculated values of each lifestyle group was compared to that of mesophiles by Dunn's multiple comparison test for Bacteria and Archaea separately. The significance levels of the distribution differences are indicated as stars (* $p < 0.05$; ** $p < 0.01$; *** $p < 0.001$; **** $p < 0.0001$). The number of proteomes in the datasets are indicated below the labels. MESO – mesophiles, PSY – psychrophiles, TH – thermophiles, HYTH – hyperthermophiles, HALO – halophiles, ALK – alkaliphiles, ACID – acidophiles, RADIO –radiotolerants, THAC – thermoacidophiles.

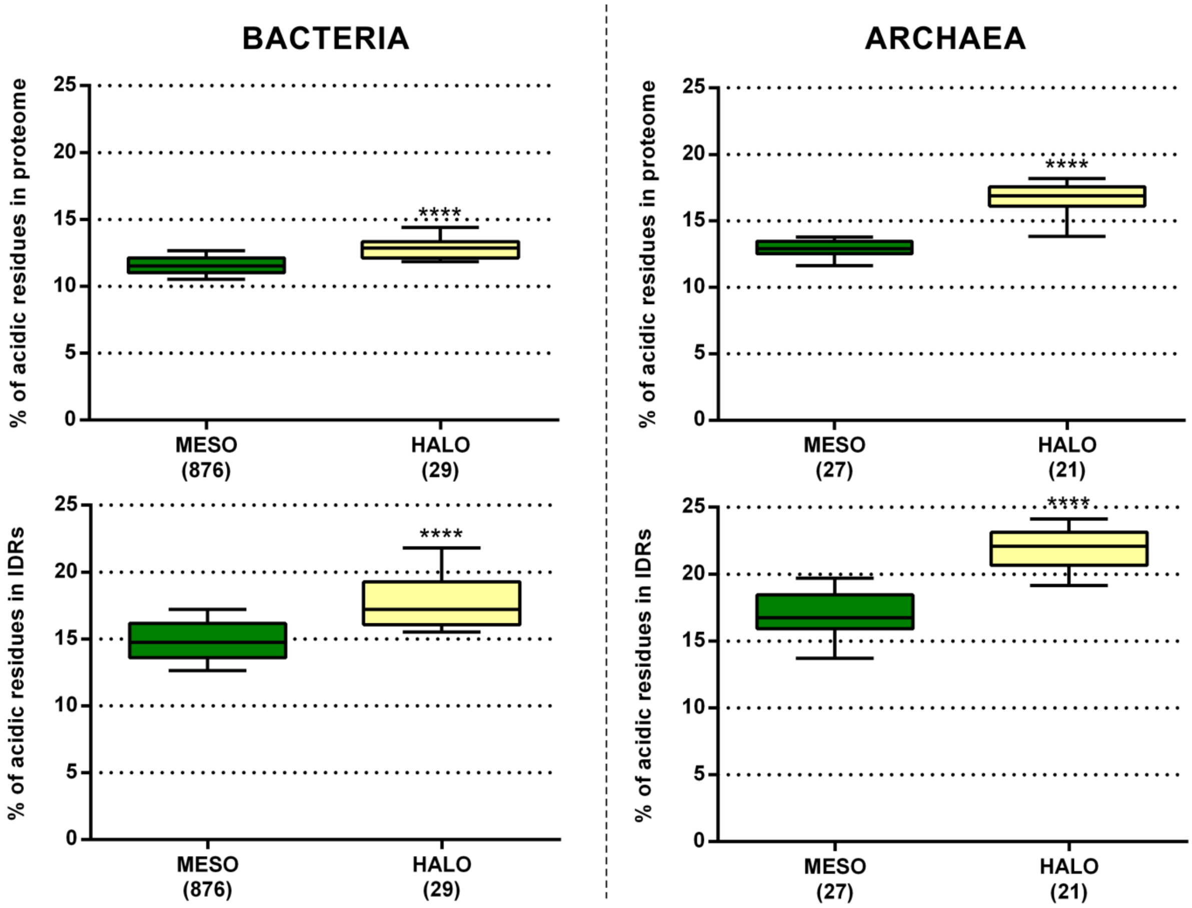

Figure S2. Fraction of acidic residues in Bacterial and Archaeal proteomes and predicted IDRs. The % of acidic residues was calculated for the proteomes and the IUPred-predicted intrinsically disordered regions (IDRs) for Bacterial and Archaeal mesophiles and halophiles. The distribution of the calculated values for halophiles was compared to that of mesophiles by Mann-Whitney U test for Bacteria and Archaea separately. The significance levels of the distribution differences are indicated as stars (**** $p<0.0001$). The number of proteomes in the datasets are indicated below the labels. MESO – mesophiles, HALO – halophiles.

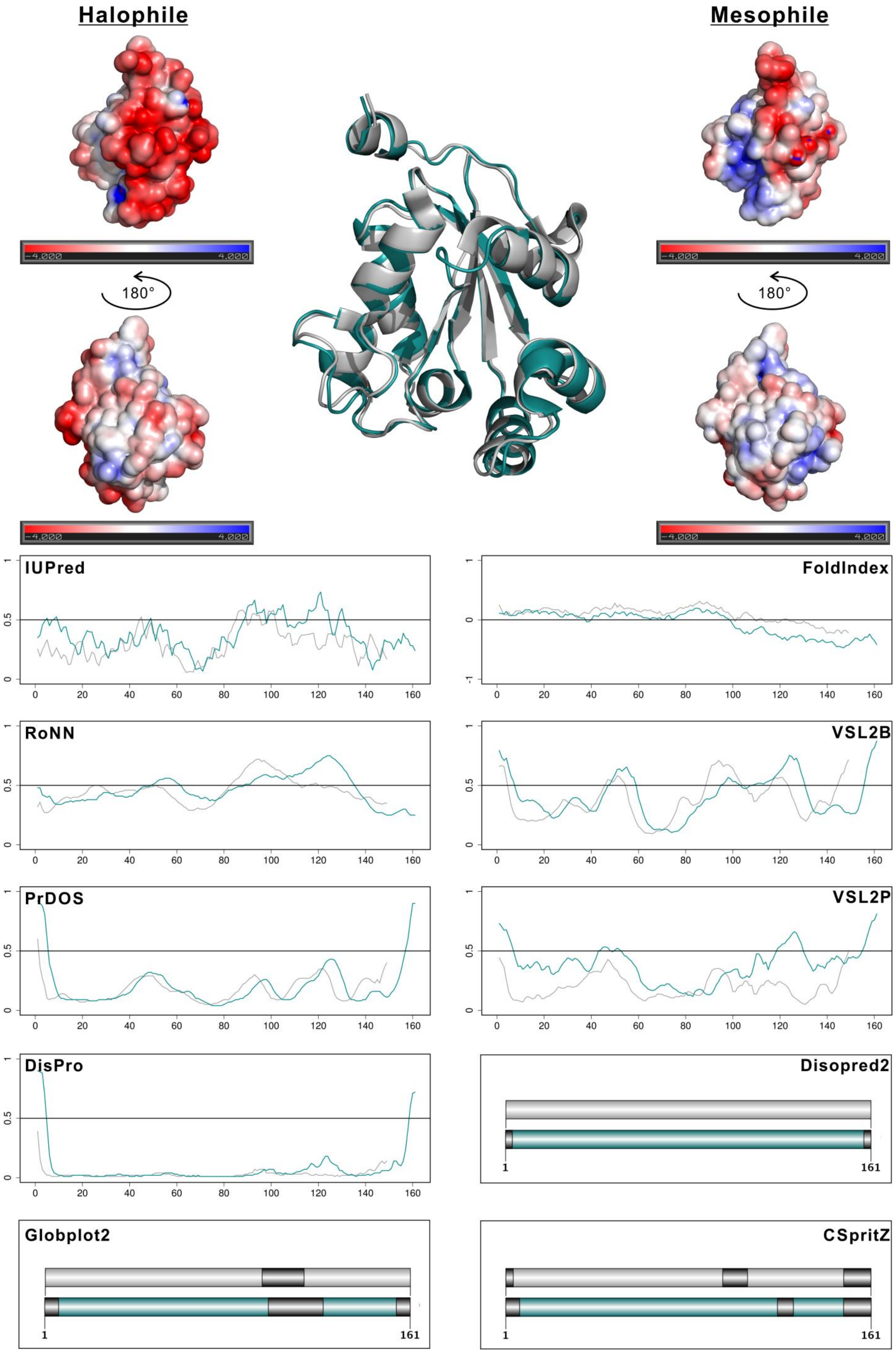

Halophile
Mesophile
-4.000
4.000
180°
IUPred
FoldIndex
RoNN
VSL2B
PrDOS
VSL2P
DisPro
Disopred2
Globplot2
CSpritZ
0
0.5
1
-1
20
40
60
80
100
120
140
160
161

Figure S3. Disorder predictions with ten methods for a halophilic and a mesophilic nucleoside diphosphate kinase enzyme. The halophilic enzyme from Halobacterium salinarum (PDB: 2AZ3) is shown on the left and with teal color in the structural alignment and within the disorder prediction plots. The mesophilic enzyme from Staphylococcus aureus (PDB: 3Q86) is shown on the right and with grey color in the structural alignment and within the disorder prediction plots. For the different disorder prediction methods, order-disorder thresholds were defined as suggested in their respective original publications. In each case values above the probability threshold mean disorder, except for FoldIndex where it is the opposite. For the seven methods that provide continuous values for disorder probability we show the predicted values as curves. The curves are continuous and just placed on each other, not fitted/aligned. Teal curves show the predictions for the halophilic, grey ones for the mesophilic enzyme. For the three methods that only provide a binary classification of residues as ordered/disordered we show the proteins as colored bars with the predicted disordered regions depicted as dark grey stripes and with the length of the halophilic protein indicated.

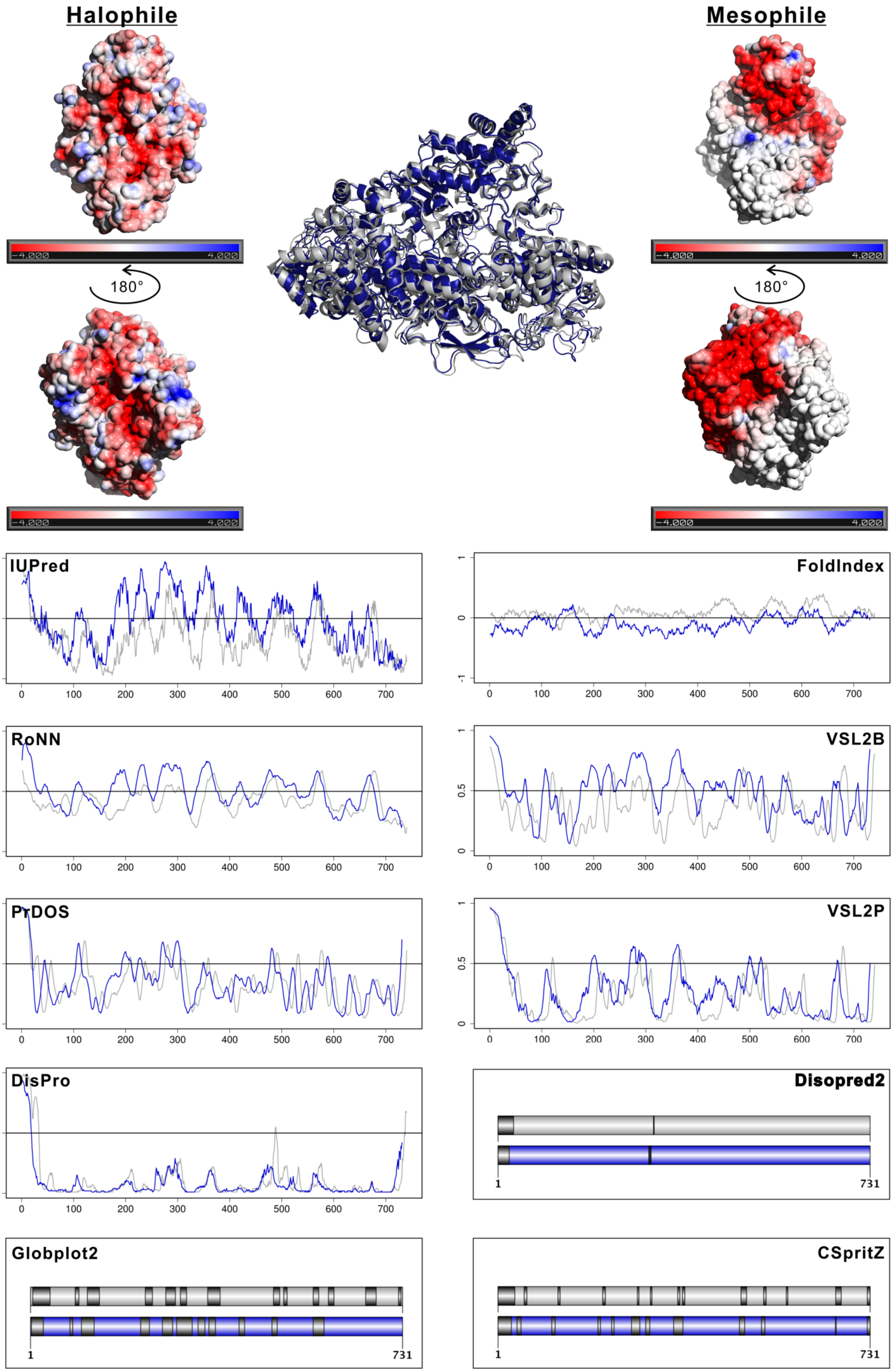

Halophile
Mesophile
-4.000
4.000
180°
IUPred
FoldIndex
RoNN
VSL2B
PrDOS
VSL2P
DisPro
Disopred2
Globplot2
CSpritZ
0
0.5
1
-1
100
200
300
400
500
600
700
731

Figure S4. Disorder predictions with ten methods for a halophilic and a mesophilic catalase peroxidase enzyme. The halophilic enzyme from Haloarcula marismortui (PDB: 1ITK) is shown on the left and with blue color in the structural alignment and within the disorder prediction plots. The mesophilic enzyme from Mycobacterium tuberculosis (PDB: 1SJ2) is shown on the right and with grey color in the structural alignment and within the disorder prediction plots. For the different disorder prediction methods, order-disorder thresholds were defined as suggested in their respective original publications. In each case values above the probability threshold mean disorder, except for FoldIndex where it is the opposite. For the seven methods that provide continuous values for disorder probability we show the predicted values as curves. The curves are continuous and just placed on each other, not fitted/aligned. Blue curves show the predictions for the halophilic, grey ones for the mesophilic enzyme. For the three methods that only provide a binary classification of residues as ordered/disordered we show the proteins as colored bars with the predicted disordered regions depicted as dark grey stripes and with the length of the halophilic protein indicated.
